# Elevated serum neurofilament light chain protein in patients with essential tremor

**DOI:** 10.1111/ene.16143

**Published:** 2023-11-17

**Authors:** Sebastian Franthal, Michael Khalil, Daniela Kern, Lukas Gattermeyer, Arabella Buchmann, Petra Katschnig‐Winter, Mariella Kögl, Rina Demjaha, Cansu Tafrali, Edith Hofer, Reinhold Schmidt, Petra Schwingenschuh

**Affiliations:** ^1^ Department of Neurology Medical University of Graz Graz Austria

**Keywords:** essential tremor, neurodegeneration, neurofilament light chain protein, pathophysiology, sNfL, tremor

## Abstract

**Background and purpose:**

Quantification of neurofilament light chain protein in serum (sNfL) enables the neuro‐axonal damage in peripheral blood to be reliably assessed and monitored. There is a long‐standing debate whether essential tremor represents a ‘benign’ tremor syndrome or whether it is linked to neurodegeneration. This study aims to investigate sNfL concentrations in essential tremor compared to healthy controls (cross‐sectionally and longitudinally) and to assess whether sNfL is associated with motor and nonmotor markers of disease progression.

**Methods:**

Data of patients with essential tremor from our prospective registry on movement disorders (PROMOVE) were retrospectively analysed. Age‐, sex‐ and body‐mass‐index‐matched healthy controls were recruited from an ongoing community‐dwelling aging cohort. sNfL was quantified by an ultra‐sensitive single molecule array (Simoa). All participants underwent detailed clinical examination at baseline and after approximately 5 years of follow‐up.

**Results:**

Thirty‐seven patients with clinically diagnosed essential tremor were included and 37 controls. The essential tremor group showed significantly higher sNfL levels compared to healthy controls at baseline and follow‐up. sNfL levels increased over time in both groups, and the slope of sNfL increase was similar in the essential tremor and healthy control groups. Comparing patients with a disease duration under 5 years to those with a longer disease duration, the former group had a significantly greater increase of sNfL over time, which strongly correlated to worsening of tremor and cognition.

**Conclusion:**

Our findings indicate that neurodegeneration, possibly happening at an early disease stage, might play a role in the pathophysiology of essential tremor.

## BACKGROUND

There is a long‐standing debate whether essential tremor (ET) represents a ‘benign’ tremor syndrome or whether it is linked to neurodegeneration especially in the cerebellum [1]. The recently released tremor classification has revised the nosology of tremor, defining ET as a syndrome that may have multiple aetiologies. Furthermore, patients with the characteristics of ET along with a resting tremor or one or more other signs of uncertain significance or relevance to tremor such as impaired tandem gait, questionable dystonic posturing or memory impairment (‘soft signs’) are now labelled as ET plus [2]. Evidence from postmortem pathological findings, neuroimaging data showing structural abnormalities in ET brains and nonmotor clinical features such as impaired cognition suggest that neurodegenerative processes might, at least to some extent, contribute to the pathophysiology of ET [1, 3, 4, 5, 6].

Neuronal and axonal destruction are hallmarks of neurodegenerative diseases. Quantification of neurofilament light chain protein in serum (sNfL) using a single molecule array (Simoa) enables neuro‐axonal damage in the peripheral blood to be reliably assessed and monitored [7]. sNfL is elevated in neurodegenerative disorders such as Alzheimer's disease, Parkinson's disease (PD) and atypical parkinsonian syndromes [8].

Quantification of sNfL may provide further insight into the underlying pathophysiology of ET and its subtypes. Postmortem electron microscope studies have revealed that torpedoes contain an excess and disorganization of neurofilaments [9]. Subsequently, normal cerebellar levels of neurofilaments but an abnormal phosphorylation state of neurofilament heavy chain were reported in 47 postmortem ET cases [10]. One cross‐sectional study found higher sNfL levels in PD compared to healthy controls (HCs) and ET, but no relevant difference between ET and HCs. Whilst sNfL was reported as an independent contributor to motor symptom and cognitive dysfunction severity in patients with PD, the relationship between sNfL and motor and cognitive function amongst patients with ET remained unclear [11]. Another recent cross‐sectional case–control study in 41 ET patients and 40 HCs revealed higher sNfL levels in ET but no correlation to clinical parameters [12].

Against this background, the aim was to investigate sNfL concentrations in ET compared to HCs (cross‐sectionally and longitudinally) and to assess whether sNfL is associated with motor and nonmotor markers of disease progression in ET.

## METHODS

### Participants

Patients were recruited from an ongoing single‐centre registry study on movement disorders (Prospective Movement Disorders Registry Graz, PROMOVE) at the Medical University of Graz, Austria, starting in 2010. Thirty‐seven patients without dementia (Mini‐Mental State Examination [MMSE] > 26 [13]) and clinically diagnosed ET [2] were included. Out of them 16 patients met the criteria for ET plus. Ten of our patients had a disease duration under 5 years with follow‐up data available for five. Thirteen patients had an age at onset over 60 years; for six of them follow‐up data were available. The majority of patients with ET (22/37) underwent single‐photon emission computed tomography imaging with DaTSCAN™ (Ioflupane 123‐I SPECT), which was reported as normal in all of them. Thirty‐seven age‐, sex‐ and body‐mass‐index‐(BMI)‐matched HCs without first‐degree relatives with any movement disorder were recruited from an ongoing community‐dwelling aging cohort [14]. Patients and controls were not accepted with a history or symptoms of dementia, head trauma, epilepsy, stroke or brain surgery. All in all, 20/37 patients and 20/37 HCs had a follow‐up visit after 4.89 ± 0.99 years and 5.00 ± 0.83 years, respectively.

All participants underwent detailed clinical examination including neurological assessment, Fahn−Tolosa−Marin Tremor Rating Scale [15] and CERAD‐Plus testing [16] to evaluate cognition. Moreover, CERAD total scores (including verbal fluency, modified Boston Naming Test, constructional praxis, word list memory, word list recall and word list discriminability for TS1 and additionally constructional recall for TS2) [17, 18] and a memory score (including word list recall, word list recognition and constructional recall) [19] were calculated. Furthermore, total scores were used for the corrected CERAD *z* scores (zTS1 including the *z* scores of the same items as TS1 and zTS2 additionally considering constructional recall and Trail‐Making Test A and B) [20]. For details see Table S1.

Brain magnetic resonance imaging scans (3 T) were performed at baseline to exclude relevant structural abnormalities including chronic vascular encephalopathy and brain atrophy.

### Standard protocol approvals, registrations and patient consents

Approval was obtained from the institutional review board of the Medical University of Graz (21‐345 ex 09/10) and all participants gave written informed consent before inclusion in this study. The study conforms with the World Medical Association Declaration of Helsinki.

### Serum neurofilament light chain protein

Blood was taken by peripheral venepuncture of all participants at baseline and follow‐up. The blood samples were centrifuged and frozen at −80°C following a standardized procedure [21]. sNfL was quantified by an ultra‐sensitive single molecule array (Simoa) on a Quanterix SR‐X Analyzer using the commercially available NF‐light® assay. To ensure inter‐assay precision all measurements were done twice; results with a coefficient of variation over 20% were excluded from our analyses.

### Statistical methods

Statistical analyses were performed using the software SPSS Statistics (IBM Statistics for Windows, Version 20) and the R statistical software version 3.6.3 (R Core Team, 2022; R: A language and environment for statistical computing. R Foundation for Statistical Computing, Vienna, Austria. URL https://www.R‐project.org/). Assumptions of normal distribution were tested with the Kolmogorov–Smirnov test. Group differences were calculated with an independent *t* test (for normally distributed variables); otherwise by Mann−Witney test. Change of sNfL over time was analysed using linear mixed‐effects models including a random effect to account for dependences due to repeated observations. Linear mixed‐effects models were calculated using the R package lmer. For clinical correlations of sNfL linear regression analyses were performed corrected for age and BMI. Due to our small number of cases in the short disease duration group, Pearson or Spearman correlations were performed to test associations in this group. Multiple comparisons were corrected with Bonferroni correction. All data are expressed as mean and standard deviation (mean ± SD). All *p* values of <0.05 were considered significant.

## RESULTS

Demographics are shown in Table 1.

**TABLE 1:** Clinical and demographic data (mean ± standard deviation).

	Essential tremor	Healthy controls
Total number	37	37
Number of participants with follow‐up visit	20	20
Follow‐up period (years)	4.89 ± 0.99	5.00 ± 0.83
Age (years)	65.28 ± 6.55	65.24 ± 7.27
Sex, *n* (male/female)	20/17	20/17
Body mass index	27.57 ± 4.51	27.30 ± 4.14
Baseline disease duration (years)	16.72 ± 16.20 (overall)	
	2.94 ± 1.69 (short disease duration group, *n* = 10)	
	21.83 ± 16.19 (remaining cohort, *n* = 27)	

**FIGURE 1:**
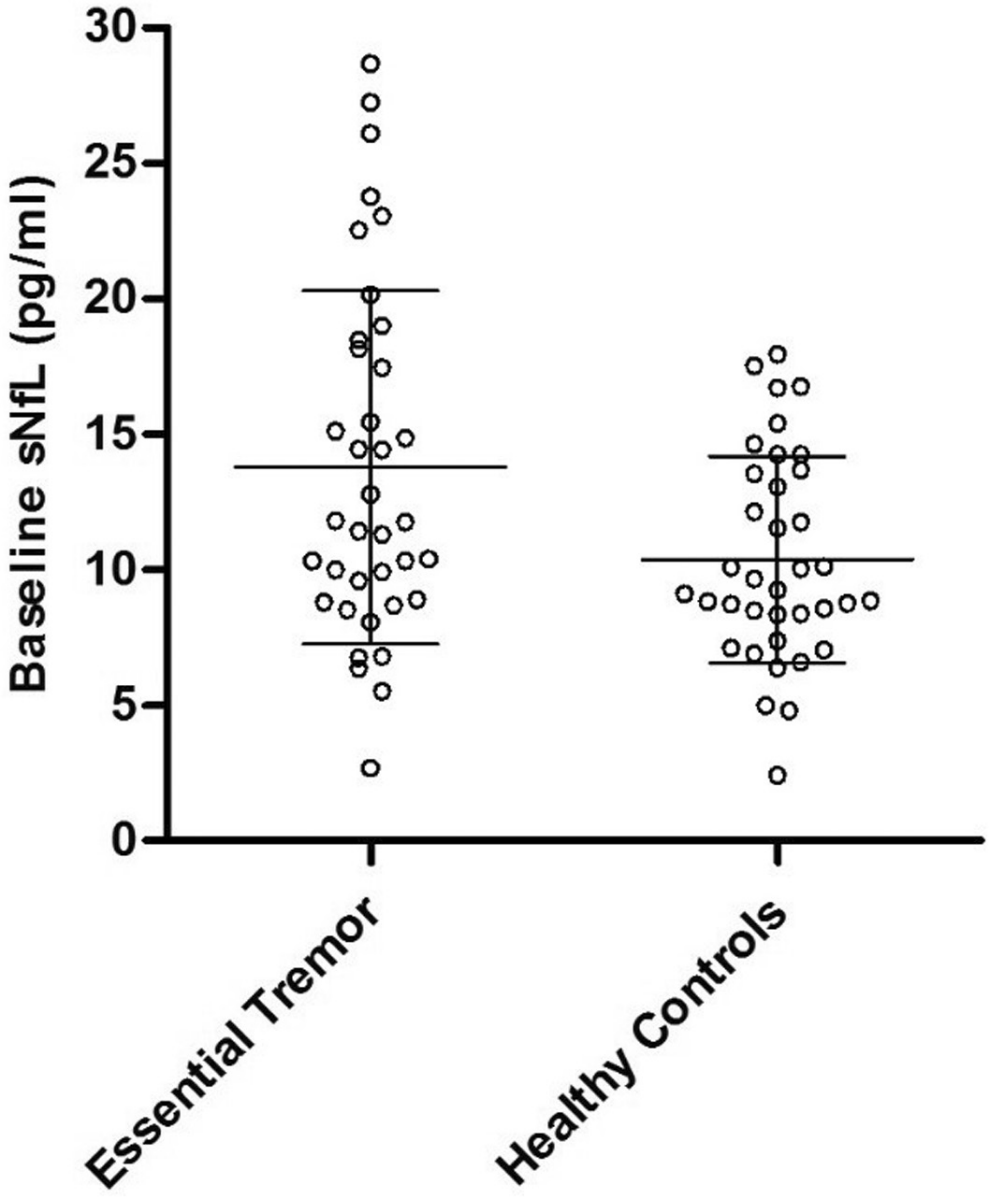
sNfL in pg/mL at baseline (median; interquartile range) in patients with essential tremor and healthy controls.

**FIGURE 2:**
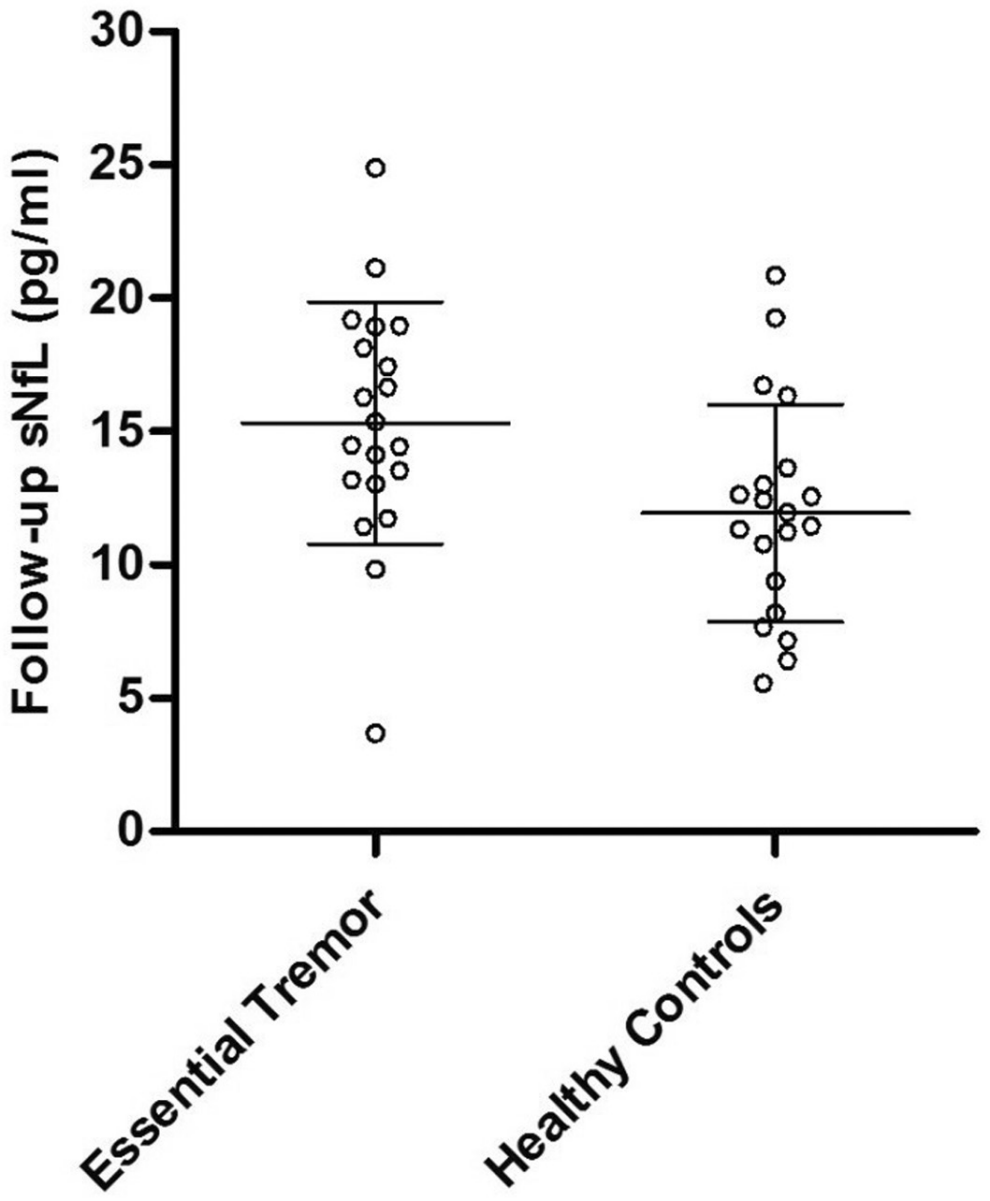
sNfL in pg/mL at follow‐up (median; interquartile range) in patients with essential tremor and healthy controls.

The ET group showed higher sNfL levels compared to HCs at baseline (ET, 13.78 ± 6.52 pg/mL; HCs, 10.38 ± 3.81 pg/mL; *p* = 0.022) and follow‐up (ET, 15.32 ± 4.53 pg/mL; HCs, 11.93 ± 4.08 pg/mL; *p* = 0.017) (Figures 1 and 2).

**FIGURE 3:**
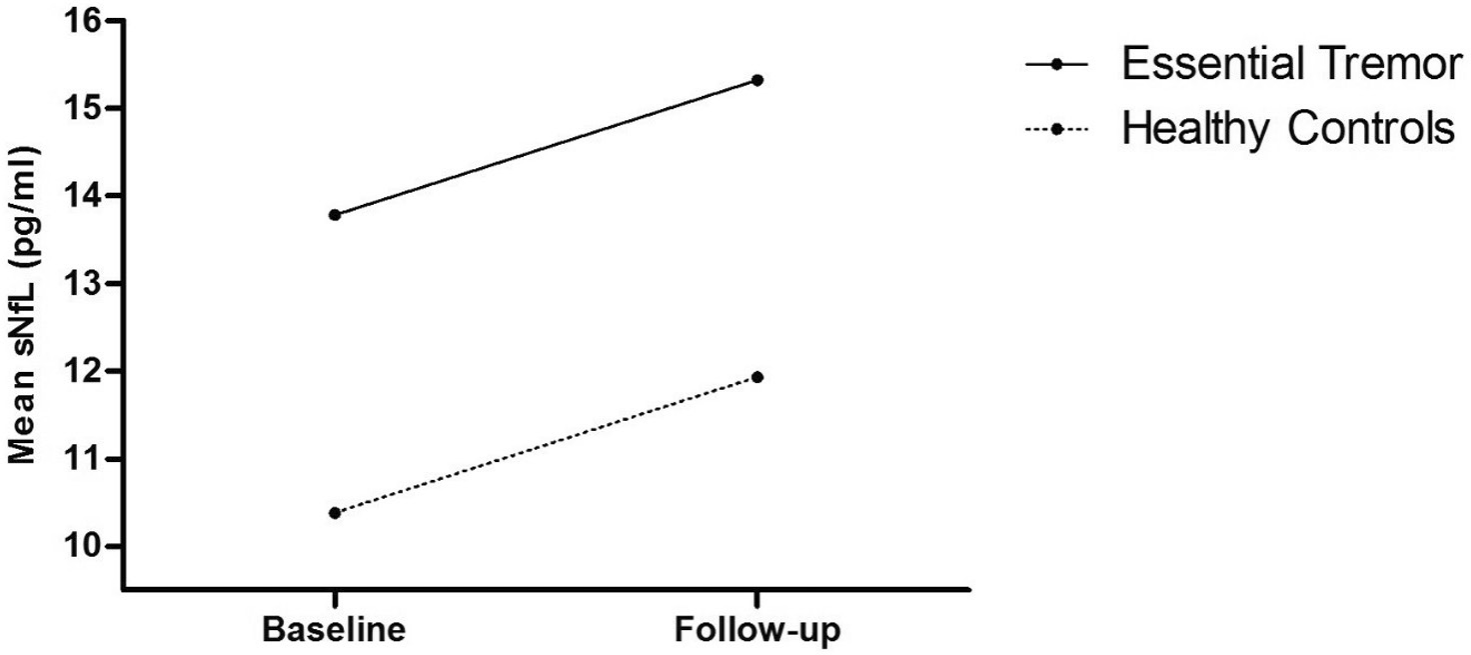
Longitudinal development of mean sNfL in pg/mL in patients with essential tremor and healthy controls.

Serum NfL levels increased over time in both groups (linear mixed‐effects model; *p* value for time 0.002), but the slope of sNfL increase was similar in ET and HCs (*p* value for time*group 0.99) (Figure 3). These results persist after adjustment for age and MMSE score (*p* value for time 0.003; *p* value for time*group 0.86).

The difference of sNfL levels at baseline and follow‐up was higher in patients with ET with a disease duration ≤5 years (short disease duration group; 5.08 ± 2.02 pg/mL) compared to those with a disease duration >5 years (long disease duration group; 0.77 ± 4.22 pg/mL; *p* = 0.004). Age did not significantly differ between these subgroups. There was no difference in sNfL change over time between patients with an age of onset under (early onset group; 1.31 ± 4.24 pg/mL) and over (late onset group; 3.08 ± 4.21 pg/mL; *p* = 0.409) 60 years.

When included as a continuous variable in the linear mixed‐effects model, neither the disease duration (*p* = 0.91) nor the age at onset (*p* = 0.56) predict the change in sNfL over time.

In our cohort, six patients had higher sNfL values at baseline than the interquartile range. All six patients had a negative DaTSCAN. These six patients were significantly older and performed weaker in cognitive tests, but—like all other patients included in the study—did not fulfil the criteria for dementia. There was no significant difference in BMI, Fahn−Tolosa−Marin Tremor Rating Scale, Non‐Motor Symptoms Questionnaire or UPSIT smelling test between these six versus the other patients. There was no difference in sNfL at baseline or follow‐up between the ET (*n* = 21) and ET plus subgroups (*n* = 16). (Baseline, ET, 14.68 ± 7.03 pg/mL; ET plus, 12.59 ± 5.77 pg/mL; *p* = 0.660. Follow‐up, ET, 15.24 ± 4.34 pg/mL; ET plus, 15.43 ± 5.03 pg/mL; *p* = 0.929.)

There is evidence suggesting a different pathophysiology in the subgroup of ET patients with head tremor, and rest tremor is a soft sign for ET plus [22, 23, 24, 25]. Therefore, logistic regression analyses were done. There was no association between sNfL and head tremor or rest tremor.

No correlation was found between sNfL at baseline and disease duration or tremor severity assessed by the Fahn−Tolosa−Marin Tremor Rating Scale.

Patients with higher levels of sNfL at baseline performed weaker in CERAD total scores corrected for age and BMI as well as the expanded CERAD zTS2 score: CERAD total score 1, beta −0.532, *p* = 0.039; CERAD total score 2, beta −0.523, *p* = 0.027; CERAD memory score, beta −0.522, *p* = 0.015; CERAD zTS1 score, beta −0.290, *p* = 0.182; and CERAD zTS2 score, beta −0.390, *p* = 0.042 [11].

To test longitudinal associations between sNfL and clinical parameters of disease progression the difference of sNfL at follow‐up and baseline was correlated to the difference of the above‐described parameters. No association was found in the total ET group. However, in the short disease duration group, the increase of sNfL was strongly associated with the increase in Fahn−Tolosa−Marin Tremor Rating Scale (Pearson's *r* = 0.907, *p* = 0.033) and to the decrease in CERAD memory score (Spearman's *ρ* = −0.949, *p* = 0.042).

To test whether sNfL at baseline might predict clinical progression of ET baseline sNfL was correlated to clinical parameters at follow‐up and the difference of follow‐up and baseline. There was no relevant association in the overall ET group and the short disease duration group.

## DISCUSSION

Our study investigates cross‐sectional and longitudinal sNfL levels in ET and therefore gives further insight in the pathophysiology of this common tremor syndrome. There are many hints that ET is not a simple benign monomorphic tremor disorder but is caused by a degenerative process in the brain. For instance, there are postmortem pathological findings and neuroimaging data showing structural abnormalities in ET brains especially in the cerebellum and basal ganglia. Moreover, nonmotor clinical features such as impaired cognition or associated movement disorders, symptom progression over time and no spontaneous remission are in line with our findings of increased sNfL as potential evidence of neurodegeneration in ET [1, 3, 4, 5, 6].

Neurofilaments are structural scaffolding proteins, exclusively expressed in neurons. Axonal destruction as a main process in neuronal cell death and consecutively neurodegeneration cause abnormally high levels of neurofilaments in the cerebrospinal fluid but also in serum. Single‐molecule arrays enable reliable quantification of low sNfL concentrations that are present in blood samples in healthy individuals. This technical advancement allows blood sNfL to be studied in healthy individuals compared to patients having several acute and chronic neurological diseases, including those conditions in which lumbar puncture as a relatively invasive procedure to collect cerebrospinal fluid is usually not performed. sNfL has been established as a marker amongst others for neurodegeneration, in stroke, posttraumatic injuries, multiple sclerosis, amyotrophic lateral sclerosis, dementia and parkinsonian disorders [8]. To our knowledge there are only two other cross‐sectional studies examining sNfL in ET with partially conflicting results. Huang et al. found only a trend for higher sNfL in ET compared to HCs whereas Salinas et al. showed significantly higher sNfL in ET. This might be due to the comparatively short disease duration or the relatively mild tremor symptoms in the cohort investigated by Huang et al. [11, 12]. Our data contribute new insights and support the growing body of research that conceptualizes ET as a syndrome with underlying neuronal destruction, but, as shown before, to a lesser extent than in PD [11]. The association between PD and ET has been broadly discussed in the literature. Evidence exists that at least in some cases clinical and pathophysiological features may overlap between these common movement disorders [26]. Increased sNfL has previously been reported in PD [8]. Thus, our findings of increased sNfL in ET may suggest some common underlying neuro‐axonal damage. This is also supported by a recent longitudinal study which showed clinical progression of motor symptoms in ET, namely spread of tremor in multiple body segments and emergence of soft signs, over time [27].

It is now well known that sNfL levels are associated with age and increase with normal aging [28]. In our longitudinal analyses a similar sNfL increase over time in HCs and ET was found. Disease duration did not predict the change of sNfL over time when included as a continuous variable in the linear mixed‐effects model, which indicates the lack of a linear association of disease duration and sNfL increase. However, neuro‐axonal damage in ET might occur as an early process in disease evolution, after which sNfL levels reach a stable level where only age‐related effects occur over time. This is supported by our finding that patients with ET with a disease duration under 5 years showed a steeper slope in sNfL increase over time compared to those with a longer disease duration.

Moreover, the increase of sNfL in early disease was strongly associated with clinical progression in the Fahn−Tolosa−Marin Tremor Rating Scale and cognitive decline in the CERAD memory score. These findings support the notion for neurodegeneration as a pathophysiological feature at least in a subgroup of patients with ET but also the slow progression and often stable state of clinical symptoms in ET compared to disorders such as PD.

Previous data suggest a more rapid disease progression in late onset ET patients [29]. Only a trend for stronger sNfL increase in late onset ET was found. This might be due to our very small late onset subgroup of only six patients and needs further investigation in a larger cohort.

No significant difference was found in sNfL in ET compared to ET plus, which might be due to the relatively small group size or indicate sNfL increase as a general process in ET independent of additional symptoms matching the criteria of ET plus.

Only patients without a diagnosis of cognitive impairment (MMSE > 26) were included. Despite this selection criterion, associations between the patients' sNfL levels and multiple domain cognitive testing by CERAD plus were found. However, prior studies showed an association between sNfL and cognitive performance in MMSE also in healthy individuals [30]. Nevertheless, the cognitive deficits, even if very mild, in association with the increased sNfL in ET might be another supporting fact of defects in different cerebral systems in ET pathophysiology.

Several limitations to our study are acknowledged. First, the number of patients investigated is relatively small. However, given the clear differences in sNfL between patients and controls it is thought that our data provide a good basis to further investigate sNfL in ET in a larger cohort preferentially in a multi‐centre study setting. ET is a very heterogeneous syndrome with various underlying aetiologies and our sample size only allows limited conclusions regarding general pathophysiological processes in ET. Secondly, the ET cohort comprised patients with a broad range of disease duration at baseline and longitudinal data on sNfL in a larger group of newly diagnosed patients with ET is clearly warranted. Moreover, several of our ET patients were on medication, namely 19 on propranolol and two on primidone, which might have influenced their cognitive performance. Finally, at the time of analysis follow‐up data were only available for 54% of the participants, which makes it difficult to draw robust conclusions on findings in subgroups such as ET with short disease duration, late onset ET or ET plus.

Altogether, our study provides evidence for an underlying degenerative process in a subgroup of patients with ET, clinically associated with subtle cognitive abnormalities and faster progression at least in the early disease stage. Our findings support the new tremor classification that regards ET as a heterogeneous syndrome rather than one disease entity [2].

Although generally regarded as progressive in nature, the course of this disease is not well understood as few longitudinal clinical studies of patients with ET are available [31]. A recent study highlighted the unpredictability of disease progression over time for many individuals with ET and the need for markers to predict the likely course of ET motor and nonmotor symptoms [32]. sNfL or probably the change of sNfL in the first years after symptom onset may be useful in subclassifying patients with ET, which is mandatory in order to uncover the so far unknown various aetiologies of this common tremor syndrome.

sNfL abnormalities have been demonstrated in several neurodegenerative diseases. Regardless of whether these protein aggregates are the cause or consequence of these diseases, sNfL abnormalities have been shown to be an important factor in the cellular disruption. Even if our sample size, especially in the early disease group, was relatively small, a probable effect of sNfL in disease development and progression of ET was shown. Therefore, further analyses of these sNfL abnormalities and their mechanisms are important to enhance our understanding of disease pathogenesis and predictors of disease progression in ET [10].

## AUTHOR CONTRIBUTIONS

**Sebastian Franthal:** Conceptualization; investigation; writing – original draft; writing – review and editing; formal analysis; methodology; validation; visualization. **Michael Khalil:** Conceptualization; methodology; validation; supervision; writing – review and editing. **Daniela Kern:** Data curation; writing – review and editing; investigation. **Lukas Gattermeyer:** Data curation; writing – review and editing; investigation. **Arabella Buchmann:** Data curation; investigation; writing – review and editing. **Petra Katschnig‐Winter:** Writing – review and editing; data curation; investigation. **Mariella Kögl:** Investigation; writing – review and editing; data curation. **Rina Demjaha:** Visualization. **Cansu Tafrali:** Visualization. **Edith Hofer:** Methodology; writing – review and editing. **Reinhold Schmidt:** Supervision; writing – review and editing. **Petra Schwingenschuh:** Conceptualization; investigation; funding acquisition; writing – review and editing; methodology; validation; project administration; data curation; supervision; resources.

## CONFLICT OF INTEREST STATEMENT

The authors declare that they have no competing interests concerning this work. Financial disclosures of all authors for the preceding 12 months are listed here: S.F. none; M.K. Grants from Bayer, Novartis, Merck, Biogen Idec and Teva Pharmaceutical Industries Ltd, Advisory boards for Biogen Idec, Merck Serono, Roche, Novartis and Gilead; D.K. none; L.G. none; A.B. Grants from Bayer, Novartis, Biogen Idec and Quanterix; P.K. Grants from Abbvie; M.K. Grants from Abbvie, Merz, UCB Pharma and Ipson; R.D. none; C.T. none; E.H. none; R.S. Advisory boards for Roche, Esai and Biogen, consultancies from Neuroscios Austria and research grants from the Austrian Alzheimer Society; P.S. Grants from Bial, Boston Scientific and Abbvie.

## ETHICS STATEMENT

The institutional review board of the Medical University of Graz, Graz, Austria, has approved the use of humans for this study (21‐345 ex 09/10).

## Supporting information


Table S1

## Data Availability

The datasets used and/or analysed during the current study are available from the corresponding author on reasonable request.
