# Supplementary material for: Elevated serum neurofilament light chain protein in patients with essential tremor
Source: Eur J Neurol. 2023 Nov 17;31(2):e16143. doi: 10.1111/ene.16143 (PMC11235714; doi:10.1111/ene.16143)
Supplement: Supplementary file 1 — Table S1 [file ENE-31-e16143-s001.docx]

|  | **Baseline** | **Follow-up** |
| --- | --- | --- |
| Fahn-Tolosa-Marin-Tremor Rating Scale | 27.86±13.46 | 27,55±14.94 |
| Mini Mental State Examination | 28.68±1.06 | 29.25±0.91 |
| CERAD total score 1 | 81.56±10.46 | 85.41±9.30 |
| CERAD total scores 2 | 91.22±11.69 | 95.55±9.76 |
| CERAD memory score | 35.94±4.22 | 37.86±3.50 |
| CERAD z-score 1 | 0.161±0.654 | 0.565±0.672 |
| CERAD z-score 2 | 0.111±0.818 | 0.476±0.574 |
| Supplementary data Table 1: Clinical test at baseline and follow-up (mean±SD) | | |
